# Simultaneous determination of perfluoroalkyl substances and bile acids in human serum using ultra-high-performance liquid chromatography–tandem mass spectrometry

**DOI:** 10.1007/s00216-019-02263-6

**Published:** 2019-11-23

**Authors:** Samira Salihović, Alex M. Dickens, Ida Schoultz, Frida Fart, Lisanna Sinisalu, Tuomas Lindeman, Jonas Halfvarson, Matej Orešič, Tuulia Hyötyläinen

**Affiliations:** 1 School of Medical Sciences, Örebro University, 702 81 Örebro, Sweden; 2 School of Science and Technology, Örebro University, 702 81 Örebro, Sweden; 3 Turku Bioscience Centre, University of Turku and Åbo Akademi University, 20520 Turku, Finland; 4 Department of Gastroenterology, Faculty of Medicine and Health, Örebro University, 702 81 Örebro, Sweden

**Keywords:** Bile acids, Human serum, LC, MS, Perfluoroalkyl substances, PFAS

## Abstract

There is evidence of a positive association between per- and polyfluoroalkyl substances (PFASs) and cholesterol levels in human plasma, which may be due to common reabsorption of PFASs and bile acids (BAs) in the gut. Here we report development and validation of a method that allows simultaneous, quantitative determination of PFASs and BAs in plasma, using 150 μL or 20 μL of sample. The method involves protein precipitation using 96-well plates. The instrumental analysis was performed with ultra-performance liquid chromatography–tandem mass spectrometry (UHPLC-MS), using reverse-phase chromatography, with the ion source operated in negative electrospray mode. The mass spectrometry analysis was carried out using multiple reaction monitoring mode. The method proved to be sensitive, robust, and with sufficient linear range to allow reliable determination of both PFASs and BAs. The method detection limits were between 0.01 and 0.06 ng mL^−1^ for PFASs and between 0.002 and 0.152 ng mL^−1^ for BAs, with the exception of glycochenodeoxycholic acid (0.56 ng mL^−1^). The PFAS measured showed excellent agreement with certified plasma PFAS concentrations in NIST SRM 1957 reference serum. The method was tested on serum samples from 20 healthy individuals. In this proof-of-concept study, we identified significant associations between plasma PFAS and BA levels, which suggests that PFAS may alter the synthesis and/or uptake of BAs.

**Electronic supplementary material:**

The online version of this article (10.1007/s00216-019-02263-6) contains supplementary material, which is available to authorized users.

**Figure:**
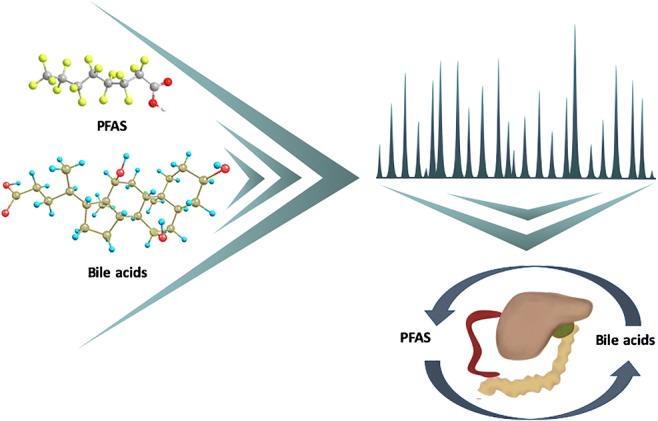
Graphical Abstract

## Introduction

In clinical studies, both in metabolomics and in environmental exposure studies, the volume of sample available for analysis is often restricted. Thus, it is desirable that the analytical methods can assay multiple substance classes simultaneously, while using as small volume of sample as possible. This presents a challenge, both regarding the chemical characteristics of the compounds that can be quantitatively covered by a single extraction and/or analytical method, as well as in terms of the concentration range of said substances which can be assayed for. While several methods have been developed for untargeted analyses which can simultaneously assay a large number of metabolites, it remains problematic to analyze simultaneously both specific metabolites and exogenous compounds (such as environmental pollutants), with the main challenge typically being the low concentrations of the latter.

In this study, we demonstrate the development of a targeted method for the analysis of two distinct compound classes, namely (i) endogenous bile acids (BAs) and (ii) exogenous per- and polyfluoroalkyl substances (PFASs) in human serum or plasma. BAs are metabolites that facilitate the digestion and absorption of lipids in the small intestine and they are also important metabolic regulators involved in the maintenance of lipid and glucose homeostasis [1, 2]. PFASs, on the other hand, are a group of man-made chemicals that have been widely used since the 1950s in both household and industrial products. PFASs also have long biological half-lives, and are readily detected in humans [3–7]. Structurally, several PFAS compounds resemble endogenous fatty acids, with fluorine substitution in place of hydrogen. Biologically, the two compound groups share some common features. It is well known that BAs that are excreted into the intestine are reabsorbed, and similar enterohepatic circulation has been suggested for PFASs [8, 9].

Recently, the European Food Safety Authority (EFSA) reported that the positive association of perfluorooctane sulfonate (PFOS) and perfluorooctanoic acid (PFOA), the two most common PFASs, with total cholesterol serum levels, as observed in several studies, may result from a possible common reabsorption of bile acids, PFOS, and PFOA from the gut and shared membrane transport pathways into the liver (https://www.efsa.europa.eu/sites/default/files/news/efsa-contam-3503.pdf). Interestingly, it has been hypothesized that 7-alpha-hydroxylase (CYP7A1), which catalyzes the first and rate-limiting step in the formation of BAs from cholesterol, may be downregulated by PFASs [9, 10]. This may lead to increased re-uptake of BAs, which would generate negative feedback loops via the farnesyl-X-receptor and subsequently reduce their de novo synthesis. Indeed, PFHxS and PFOS have been shown to decrease fecal BA excretion [11]. Thus, current knowledge already suggests concomitant reabsorption of BAs and PFASs in the intestine and that this could play a role in the observed association between serum levels of PFAS and cholesterol. At present, there is, however, limited empirical data demonstrating such a relationship.

Several methods have been developed for the quantitative determination of BAs and PFASs, using separate analytical methods [12–14]. Most of the methods include sample preparation using either (i) protein precipitation, often combined with further clean-up steps (e.g., phospholipid removal), particularly for PFASs, (ii) liquid–liquid extraction, or (iii) solid-phase extraction. Such analyses are performed predominantly with various LC–MS/MS methods, typically using reversed-phase LC in combination with triple quadrupole MS in multiple reaction monitoring mode. For PFAS analyses, the sample volumes required for analysis are typically several hundreds of microliters, while for BAs, smaller volumes are typically sufficient.

In this study, the aim was to develop and validate a quantitative method covering both BAs and PFASs in a single analysis, minimizing the required volume of serum. The method was validated by analysis of serum samples from healthy subjects, where the associations between PFAS and BA levels were investigated.

## Experimental

### Chemicals

Ammonium acetate (NH_4_Ac) was obtained from Sigma–Aldrich (St. Louis, USA). Methanol (MeOH) and acetonitrile (ACN) (both HPLC grade with purity greater than 99%) were obtained from Fisher Scientific UK (Loughborough, UK). Acetonitrile (Optima® LC–MS grade) and formic acid (98–100%) were purchased from Sigma–Aldrich (Steinheim, Germany). The MilliQ water used to make the mobile phase was 18.2 MΩ. LC vials and OstroTM 96-well plate (25 mg 1/pkg) were purchased from Waters (Waters Corporation, Milford, USA). Newborn bovine serum (New Zealand) was purchased from Sigma–Aldrich and stored frozen (≤ − 20 °C) until analysis. For quality assurance (QA), standard reference material serum SRM 1957 was purchased from the National Institute of Standards and Technology (NIST) at the US Department of Commerce (Washington, DC, USA). The SRM sample was stored frozen (≤ − 20 °C) until analysis. The quality control (QC) reference sample consisted of pooled human plasma collected from blood donors at Örebro University Hospital (Örebro, Sweden), and stored frozen (≤ − 20 °C) until analysis.

Abbreviations of target analytes are presented in Table 1. ^13^C-labeled PFAS internal standards (IS), ^13^C-labeled performance standards, and native calibration standards (perfluorocarboxylic acids (PFCAs) and perflurosulfonic acids (PFSAs)) were purchased from Wellington Laboratories (Guelph, Ontario, Canada). CA, CDCA, DCA, DHCA, GCA, GCDCA, LCA, TCA, TCDCA, TDCA, TDHCA, THCA, THDCA, TLCA, and TUDCA were obtained from Sigma–Aldrich (St. Louis, MO, USA); HDCA, HCA, αMCA, βMCA, ωMCA, 7-oxo-HDCA, 7-oxo-DCA, 12-oxo-LCA, TαMCA, TβMCA, TωMCA, GDHCA, GHCA, and GHDCA from Steraloids (Newport, RI, USA); GLCA and GUDCA from Calbiochem (Gibbstown, NJ, USA); and GDCA and UDGA from Fluka (Buchs, Switzerland). Internal standards CA-d4, LCA-d4, UDCA-d4, CDCA-d4, DCA-d4, GCA-d4, GLCA-d4, GUDCA-d4, and GCDCA-d4 were obtained from Qmx Laboratories Ltd. (Essex, UK). All standards were prepared in methanol and stored refrigerated (4 °C).

**Table 1:** Abbreviations of analytes measured

Full name	Abbreviation
Chenodeoxycholic acid	CDCA
Cholic acid	CA
Deoxycholic acid	DCA
Glycochenodeoxycholic acid	GCDCA
Glycocholic acid	GCA
Glycodeoxycholic acid	GDCA
Glycoursodeoxycholic acid	GUDCA
Lithocholic acid	LCA
Taurochenodeoxycholic acid	TCDCA
Taurocholic acid	TCA
Taurodeoxycholic acid	TDCA
Taurolithocholic acid	TLCA
Tauroursodeoxycholic acid	TUDCA
Ursodeoxycholic acid	UDCA
12-Oxolithocholic acid	12-oxo-LCA
7-Oxodeoxycholic acid	7-oxo-DCA
7-Oxohyocholic acid	7-oxo-HCA
β-Muricholic acid	βMCA
3α,7α-Dihydroxycholestanoic acid	DHCA
Glycodehydrocholic acid	GDHCA
Glycohyocholic acid	GHCA
Glycohyodeoxycholic acid	GHDCA
Glycolithocholic acid	GLCA
Hyocholic acid	HCA
Hyodeoxycholic acid	HDCA
α,β-Tauromuricholic acid	TαβMCA
Taurohyodeoxycholic acid	TDHCA
Taurodeoxycholic acid	THCA
Taurohyodeoxycholic acid	THDCA
ω-Tauromuricholic acid	TωMCA
ω-Tauromuricholic acid	ωαMCA
Perfluorobutanoic acid	PFBA
Perfluorobutane sulfonate	PFBS
Perfluorodecanoic acid	PFDA
Perfluorododecanoic acid	PFDoDA
Perfluorododecane sulfonate	PFDoDS
Perfluorodecane sulfonate	PFDS
Potassium perfluoro-4-ethylcyclohexanesulfonate	PFECHS
Perfluoroheptanoic acid	PFHpA
Perfluoroheptane sulfonate	PFHpS
Perfluorohexane sulfonate	PFHxS
Perfluorononanoic acid	PFNA
Perfluorononane sulfonate	PFNS
Perfluorooctanoic acid	PFOA
Linear-perfluorooctane sulfonate	L-PFOS
Perfluorooctane sulfonamide	PFOSA
Perfluoropentanoic acid	PFPeA
Perfluoro pentane sulfonate	PFPeS
Perfluorotetradecanoic acid	PFTDA
Perfluorotridecanoic acid	PFTrDA
Perfluoroundecanoic acid	PFUnDA

### Samples

The serum samples (*n* = 20) were from blood donors at ≥ 55 years of age, without any gastrointestinal disease, obtained from Örebro University Hospital. The samples were collected and registered between years 2012 and 2014. All the participants gave written consent. All serum samples were stored at − 80 °C until analysis. Demographic characteristics of the study sample are shown in Electronic Supplementary Material (ESM) Table S1.

### Sample preparation

The sample preparation procedure was performed as follows: all glassware and analytical syringes used were thoroughly rinsed with methanol (three times). Ten microliters of PFAS internal standard mixture (*c* = 200 ng mL^−1^ in methanol; ESM Table S2) and 20 μL of BA internal standard mixture (*c* = 440–670 ng mL^−1^ in methanol; ESM Table S2) and 150 μL serum or plasma were added to a 25-mg Ostro Protein Precipitation and Phospholipid Removal 96-well plate (Waters Corporation, Milford, USA), pre-conditioned with 450 μL acetonitrile. A 450-μL aliquot of acetonitrile (containing 1% formic acid) was added to all wells and mixed thoroughly with the sample by aspirating three times using an automated pipette. Samples were extracted using a 10″ vacuum manifold for between 5 and 7 min. Aliquots of 600 μL of the eluate from each collection plate insert were then transferred to glass LC vials and evaporated to ca. 190 μL using nitrogen. ^13^C-performance standards were added (10 μL of 200 ng mμL^−1^ PFAS in methanol; ESM Table S2) as was 300 μL of 2 mM NH_4_AC in water. All samples and standards were ultrasonicated for 10 min prior to instrumental analysis to ensure homogeneity. Samples that showed precipitation were centrifuged at 9900 min^−1^ for 10 min. In addition, we tested the procedure with 20 μL of serum, using the same internal standard mixtures and overall procedure, with two exceptions: (i) a frit filter plate was used which did not remove phospholipids (96-Well Protein Precipitation Filter Plate, Sigma–Aldrich) and (ii) after elution, the solvent was evaporated to dryness and the residue was dissolved in 20 μL of a 40:60 MeOH to H_2_O v/v mixture containing the same ^13^C/PFAS performance standards as the 150-μL method.

### Method calibration curve

Matrix-matched calibration standards were made using newborn bovine serum. In order to facilitate simultaneous analysis and subsequent quantification of PFASs and BAs, the newborn bovine serum was first filtered through an activated charcoal cartridge. Activated charcoal purification has previously been shown to efficiently remove planar molecules, including sterols, from biological samples [15] and was used to remove BAs present in high concentrations in the newborn bovine serum. Briefly, 3 mL of newborn bovine serum was filtered through an ENVI-Carb SPE cartridge (250 mg) (Sigma–Aldrich, Steinheim, Germany) and used for subsequent calibration curves.

#### Matrix-matched calibration curves for PFASs

The standards were prepared by spiking 150 μL of pretreated newborn bovine serum with the native standard mixture resulting in an 8-point matrix-matched curve ranging from 0.02 to 60 ng mL^−1^, including the matrix blank. The matrix-matched standards were further treated in the same way as authentic samples.

#### Matrix-matched calibration curves for BAs

The standards were prepared by spiking 150 μL of pretreated newborn bovine serum with the native standard mixture resulting in an 8-point matrix-matched curve ranging from 1 to 320 ng mL^−1^, including the matrix blank. The matrix-matched standards were further treated in the same way as authentic samples.

### LC–MS analysis

Analyses were performed on an Acquity UPLC system coupled to a triple quadrupole mass spectrometer (Waters Corporation, Milford, USA) with an atmospheric electrospray interface operating in negative ion mode. Aliquots of 10 μL of samples were injected into the Acquity UPLC BEH C18 2.1 mm × 100 mm, 1.7-μm column (Waters Corporation). A trap column (PFC Isolator column, Waters Corporation) was installed between the pump and injector and used to retain fluorinated compounds originating from the HPLC system and the mobile phase. The eluent system consisted of (A) 2 mM NH_4_Ac in water and (B) methanol (9:1) and 2 mM NH_4_Ac in methanol. The gradient was programmed as follows: 0–1 min, 1% solvent B; 1–13 min, 100% solvent B; 13–16 min, 100% solvent B; 16–17 min, 1% solvent B, flow rate 0.3 mL/min. The total run time for UPLC-MS/MS analysis was 17 min, while the total run time for each sample injection was 20 min, including the reconditioning of the analytical column.

MS analysis was performed in multiple reaction monitoring (MRM) mode and experimental details of the MS/MS method are given in ESM Table S2. The cone and collision energies were optimized for each analyte along with the precursor ion and product ion (*m*/*z*) which is shown with the abbreviations in ESM Table S2. Monitoring of the transitions between molecular anion [M–H]^−^ for the PFCAs and PFSAs and one product ion; [M–COOH]^−^ and [FSO_3_]^−^ were used for quantification of PFCAs and PFSAs, respectively. Additional 1–2 product ions were monitored as qualification ions except for PFPeA and PFHxA, for which only one product ion was monitored. For BAs, monitoring of the transitions between molecular anion [M–H]^−^ and one to two product ions including [SO_3_]^−^, [taurine–H]^−^, [CH_2_CHSO_3_]^−^, and [NH_2_CH_2_COO]^−^ for the conjugated BAs and [M–H]^−^ and [M–H–2H_2_O]^−^ for the non-conjugated BAs.

### Method validation

For method validation, the following parameters were investigated: linearity, method detection limit (MDL), repeatability, accuracy and precision, recovery, and matrix effect. Linearity was determined with matrix-matched standards, and plotted as relative peak areas (analyte/internal standard) versus analyte concentration.

## Results and discussion

**Fig. 1:**
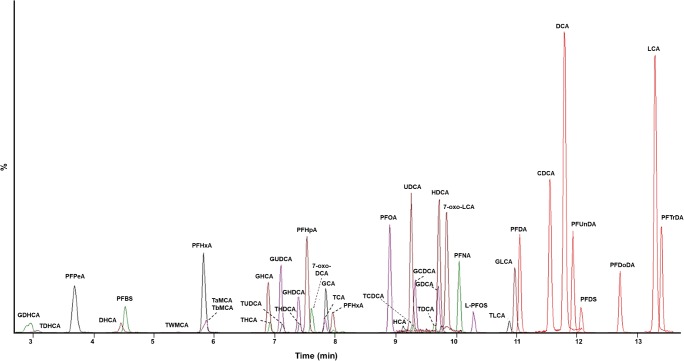
Example extracted ion chromatogram of target analytes in elution order, in accordance with ESM Table S2

The analytical method was a combination of two methods that we developed earlier for BAs and PFASs [12, 16]. The main aim was to simultaneously determine both compound classes to enable human PFAS exposure studies to investigate a potential interaction of serum/plasma PFAS and BA concentrations (Fig. 1). The method was validated for both 150 μL and 20 μL of serum/plasma.

### Validation

#### Specificity

The method provided good chromatographic robustness, with the deviation of retention times within a time margin of ± 2.5%. For the majority of compounds, multiple product ion fragments were monitored. In the case of the known co-eluting compounds, at least two product ions from the specific precursor ion were monitored with the requirement that the ion ratio for the secondary product ion be within 50% variation from the selected quantification reference. For example, in the case of L-PFOS and TCDCA and TDCA (including TUDCA and THDCA) (which share the same mass of product ion *m*/*z* = 499 and major fragment at *m*/*z* = 80 and tend to co-elute), we specifically monitored two different (other than 499 > 80) product ions unique to the two compounds in order to ensure interference-free quantitation (ESM Fig. S1).

#### Linearity

The linearity (*R*^2^) of the matrix-matched calibration curves ranged from 0.9995 to 0.99998 for PFASs and from 0.9906 to 0.9997 for BAs. The relative response factors (RRFs) of the calibration curve ranged between 0.603 and 1.47 for PFASs and between 0.23 and 1.70 for BAs with relative standard deviation < 15% for all major serum PFASs and < 22% for the broad range of BAs with the exception of three BAs above 25%: CA (26%), HCA (27%), and ωαMCA (31%).

#### Sensitivity

MDLs were determined by calculating the mean concentrations plus three times the standard deviation in water blanks run with the sample batch (*n* = 7). Overall, the method performed well, giving MDLs in the range of 0.01–0.06 ng mL^−1^ for PFAS using 150 μL of sample. The MDLs for BAs were in the range of 0.002–0.152 ng mL^−1^, with the exception of CA (0.1 ng mL^−1^) and GCDCA (0.56 ng mL^−1^), which gave higher MDLs due to higher background noise in the water blanks. With a 20-μL sample volume, the MLDs were in the range of 0.02–0.5 ng mL^−1^ for all PFASs except for PFUnDA, for which the MDL was 10 ng mL^−1^. For BAs, the MLDs were in the range of 0.0025 to 1.0 ng mL^−1^ with the exception of wMCA and 12-oxo-LCA which had higher MDL (10 ng mL^−1^).

#### Accuracy and precision

**Fig. 2:**
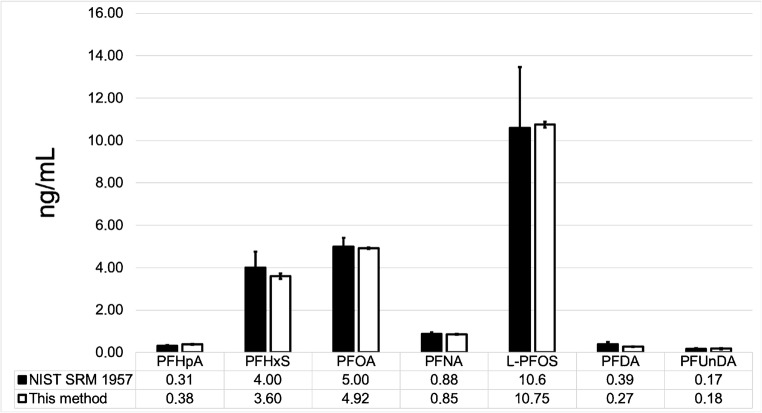
Conformity of measured PFAS concentration with certified values in NIST SRM 1957. 95% confidence intervals (CI) are (i) based on 4 replicates (our method) or (ii) based on published CI (NIST SRM 1957) [32]

The method provided accurate data and conformed well to certified serum PFAS concentrations of NIST SRM 1957. The method was reproducible with relative standard deviations (*n* = 10, NIST and in-house QC plasma), ranged from 2 to 7% for all major plasma PFAS, with the exception of PFUnDA at 24% in the NIST sample (Fig. 2, ESM Table S3). The compounds had a lower recovery, most likely due to matrix effects in that sample, as the recovery and repeatability were clearly better in the in-house QC sample. We are unaware of any NIST SRM–certified values for BAs and thus performed plasma spike tests at four five levels, 0, 50, 100, 200, and 400 ng/mL of 35 BAs. The matrix spike test results demonstrated our ability to detect BAs within an error range of 20%, with recoveries ranging from 79 to 106% for most BAs (ESM Table S4). We also evaluated potential matrix ionization enhancement or suppression using nine internal BA standards and the PFAS performance standards which were added to extract prior to injection both to determine the internal standard recovery and to monitor the trending instrument performance at two concentration levels, 50 and 100 ng/mL (ESM Fig. S2). We did not observe any deviations, other than those expected at 25% difference. Similar differences were observed for PFASs. Taken together, we can conclude that the method enables simultaneous analysis of PFASs and BAs. We also analyzed ten pooled serum samples using 20 μL of sample, and the average RSDs for both the PFAS and BA were < 10% (ESM Table S5). However, some of the PFASs and BAs were below the MLD of the method. Overall, it is possible to reduce the sample volume, however, with some compromises in the sensitivity of the method.

Comparing the here-presented combined PFAS and BA method to the previously reported separate methods for these two groups of compounds, the novel method allowed us to minimize the sample consumption, while allowing faster analysis with similar sensitivity, linear range, and accuracy. Since it was possible to reduce the sample volume down to 20 μL, without substantial loss of sensitivity or accuracy, our approach is a viable option when the sample volume is limited. However, if the goal is to perform a comprehensive determination of BA and PFAS profiles, the use of larger sample volume is recommended. Methods developed for PFASs typically report LOD/LOQ values ranging from 0.018 to 0.15 ng/ML [13, 16, 14], and for BAs in the range of 0.1 to 50 ng/mL [12, 17, 18], which are very similar to our values obtained, when using the 150-μL extraction. As demonstrated with the analysis of the certified NIST serum sample, our reported method was highly accurate for PFAS. For BAs, no certified material was available; however, the spiked samples gave robust results. Here, we have used matrix-matched calibration for PFAS and BA after matrix purification. Application of the matrix-matched calibration for PFAS analyses has improved precision; however, since the internal standards are applied in the quantitation, the impact of the possible matrix suppression would not significantly hamper the accuracy of the method substantially, as has been shown also in inter-laboratory studies [19]. In our study, based on the evaluation of the matrix effects (ESM Fig. S2), the effect was in average < 20%. Thus, application of a single calibration mixture of pure standards is, in principle, a feasible option, without a major impact on the precision of quantitation.

### PFASs and BAs in human serum

The median concentrations of the PFASs and BAs in a series of 20 samples from healthy human individuals are shown in Table 2. Of the 20 measured PFASs, seven PFASs could be detected in a majority of the samples, and 19 bile acids were detected in > 70% of the samples, and these were taken for further data analysis. In the measured set of samples, the age or the sex did not have a significant impact on the measured concentrations.

**Table 2:** Measured concentration values from 20 healthy individuals

	Median concentration (ng/mL)	Min. (ng/mL)	Max. (ng/mL)
CA	30.21	5.14	537.29
CDCA	80.11	9.87	606.25
GCA	254.40	64.07	1039.72
GCDCA	761.93	90.22	2113.96
TCDCA	97.92	11.39	371.25
12-oxo-LCA	13.26	2.45	34.82
DCA	240.82	0.01	737.10
HDCA	86.99	8.48	498.08
LCA	6.73	0.00	21.96
UDCA	30.12	16.76	194.31
GDCA	295.31	0.14	2154.80
GHCA	5.66	2.50	15.89
GHDCA	7.60	0.05	46.41
GLCA	20.32	4.25	141.00
GUDCA	34.94	4.49	264.67
TαβMCA	1.97	0.00	17.94
TDCA	35.69	2.37	113.93
THCA	1.60	0.00	7.71
TLCA	3.19	1.09	12.60
PFHxS	0.78	0.07	6.18
PFOA	1.42	0.15	3.48
PFNA	0.76	0.06	2.03
L-PFOS	4.20	0.44	16.69
PFDA	0.37	0.04	0.84
PFUnDA	0.39	0.06	0.91
PFTrDA	0.08	0.01	0.20

**Fig. 3:**
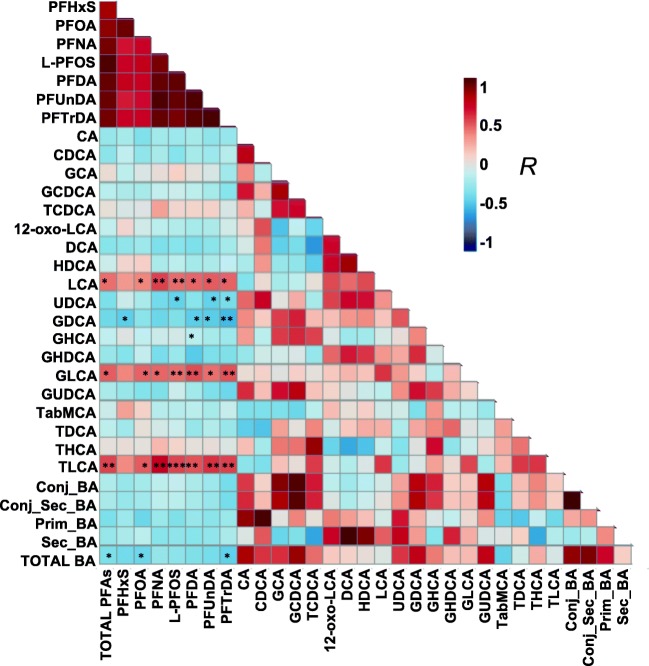
Correlation plot of PFASs and BAs (Spearman’s correlation), significance of the correlations is marked (***p < 0.01;**p < 0.05; *p < 0.1)

Next, we studied the correlation between PFAS and BA concentrations. As can be seen in Fig. 3, specific bile acids, namely LCA, GDCA, GLCA, and TLCA, showed significant associations with PFAS concentrations, with negative association between GDCA and positive associations between lithocholic acids and its two conjugates (GLCA and TLCA). The overall trend, although not reaching statistically significant in all compounds, was that the majority of circulating BAs were negatively associated with PFAS. This would suggest that the de novo synthesis of BAs is downregulated, in accordance with the literature. Specifically, our findings are in line with a previous report, where several PFASs were found to suppress CYP7A1, an enzyme that controls the first and rate-limiting step in the formation of BAs from cholesterol [11].

**Fig. 4:**
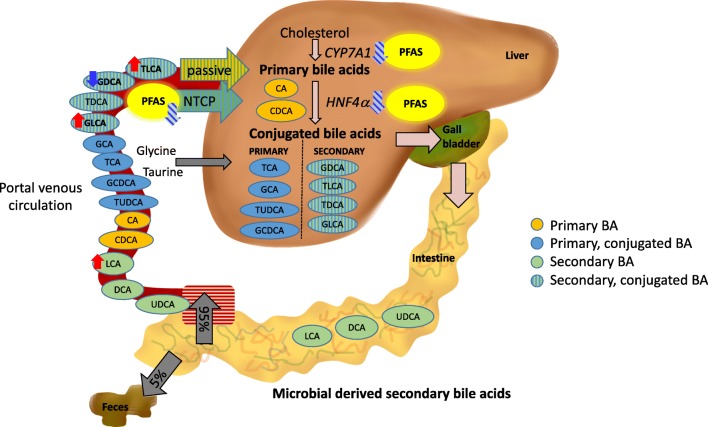
The enterohepatic circulation of bile acids. Primary bile acids (CA, cholic acid; CDCA, chenodeoxycholic acid) are synthetized from cholesterol in the liver, with the first step controlled primarily via the action of cholesterol 7α-hydroxylase (CYP7A1) which is downregulated by PFAS. Before the primary bile acids are secreted into the canalicular lumen, they are conjugated with either of the amino acids, glycine or taurine. HNF4α can regulate the genes involved in BA biosynthesis, including hydroxylation and side chain β-oxidation of cholesterol in vivo. Once in the large intestine, bacterial flora catalyzes their biotransformation into secondary bile acids: deoxycholic acid (DCA) and lithocholic acid (LCA). Ursodeoxycholic acid (UDCA) derives from epimerization of CDCA. From the colon, around 95% are reabsorbed into the distal ileum. The absorbed primary and secondary bile acids and salts are transported back to the liver where most of the conjugated BAs as well as PFASs are actively transported into hepatocytes by sodium (Na+)-taurocholate co-transporting polypeptide (NTCP). Once in the liver, the BAs are reconjugated and then re-secreted together with newly synthesized bile salts. Red arrows, positive association with PFAS; blue arrow, negative association with PFAS. The impacts on CYP7A1, HNF4a, and NTCP are based on the literature [10, 8, 21, 22].

The increased levels of LCA and its two conjugated BAs, on the other hand, could indicate any of (i) increased re-uptake of the bile acids in the gut, (ii) decreased clearance from the blood, (iii) increased production of the conjugated bile acids in the liver, or (iv) decreased de-conjugation of them by the microbiota, or any combination of these. Additionally, our results appear to suggest increased microbial formation of LCA. Indeed, PFAS exposure has been shown to cause alteration in gut microbiota, with higher exposure to PFAS associated with reduced microbiome diversity [20]. On the other hand, the decreased fecal BA excretion, linked with PFAS exposure [11], may be nonexclusively due to inhibition of the biosynthesis of the BAs [8, 21, 10] or due to the increased uptake of BAs in the gut. It has been shown that PFOA inhibits the function of the hepatocyte nuclear factor 4α [22], which plays a central role in the regulation of BA metabolism in the liver, and is linked both with the synthesis and conjugation of primary BAs. Overall, there was a negative association between conjugated BAs and PFASs, and thus, it is more likely that the observed increase of the two conjugated BAs is related to either their re-uptake or decreased de-conjugation (Fig. 4). The liver clears most of the BAs via sodium taurocholate co-transporting polypeptide (NTCP), which has a high affinity for all conjugated BAs, while the unconjugated BAs, such as LCA, are using mainly passive transport [23]. Interestingly, it has been demonstrated that PFBS, PFHxS, and PFOS are also substrates for human NTCP [8]. However, as LCA is transported passively, the changes observed are more likely due to the increased uptake from the gut than due to increased clearance. Interestingly, LCA, but not the other BAs, is an activator of nuclear receptor vitamin D receptor (VDR), which in turn regulates the intestinal barrier functions. It has been suggested that activation of VDR may be involved in increasing BA absorption and in suppressing hepatic BA synthesis [24–26].

Taken together, our observed associations between PFASs and BAs are potentially important for our understanding of cardiometabolic diseases, which is because BA metabolism is known to play a role in the pathogenesis of type 2 diabetes (T2D), atherosclerosis, and non-alcoholic fatty liver disease (NAFLD) [27]. Taurine-conjugated BAs have, for example, been found to be elevated in T2D [28]. Moreover, LCA can be cytotoxic, leading to oxidative stress, membrane damage, and colonic carcinogenesis [29], while TLCA is known to induce cholestasis by impairing biliary BA secretion [30, 31].

## Conclusions

The method presented here is suitable for fast, automated analysis of PFASs and BAs from human serum, and the sample amount can be reduced to 20 μL, however, with some loss of sensitivity. Our validation of the method demonstrated that the method is robust and accurate. Our preliminary findings that various PFASs have significant association with BAs support the notion that they may play a role in the health impacts of PFAS exposure, such as the known impact of PFAS on cholesterol levels, and in metabolic pathologies such as T2D and NAFLD. Taken together, our findings warrant further investigation of the impact of both specific and mixtures of PFAS on BA metabolism, including the potential role of gut microbiota. Such studies may provide valuable insight into the pathogenesis and varying incidence of common metabolic and immune-mediated inflammatory disorders.

## Electronic supplementary material

ESM 1 (PDF 419 kb).
