# Supplementary material for: Simultaneous determination of perfluoroalkyl substances and bile acids in human serum using ultra-high-performance liquid chromatography–tandem mass spectrometry
Source: Anal Bioanal Chem. 2019 Nov 23;412(10):2251–9. doi: 10.1007/s00216-019-02263-6 (PMC7118038; doi:10.1007/s00216-019-02263-6)

Analytical and Bioanalytical Chemistry

Electronic Supplementary Material

Simultaneous determination of perfluoroalkyl substances and bile acids in human serum using ultra-high-performance liquid chromatography-tandem mass spectrometry

Samira Salihović, Alex M. Dickens, Ida Schoultz, Frida Fart, Lisanna Sinisalu,
Tuomas Lindeman, Jonas Halfvarson, Matej Orešič, Tuulia Hyötyläinen

Table S1 Demographic characteristics of the study population (n=20)

Parameter	Median (range)
**Age (years)**	58.5 (55-69)
**Sex**	13/7(Male/Female)

Table S2 Acquisition parameters including the list or targets compounds ordered by retention time

	Target analytes	Abbreviation	Retention time	Precursor Ion (m/z)	Product Ion (m/z)		
					1	2	3
1	Glycodehydrocholic acid	GDHCA	2.97	458.1	74.0	348.1	388[illegible]
2	Taurodehydrocholic acid	TDHCA	3.09	508.1	80.0	106.9	124[illegible]
3	Perfluoropentanoic acid	PFPeA	3.70	262.7	68.9	219.0	
4	Dihydroxycholestanoic acid	DHCA	4.45	401.1	215.0	249.0	331[illegible]
5	Perfluorobutane sulfonate	PFBS	4.53	298.9	80.0	98.9	
6	Tauro-omega-muricholic acid	TwMCA	5.38	514.2	80.0	106.9	123[illegible]
7	Perfluorohexanoic acid	PFHxA	5.82	312.8	68.9	119.0	269[illegible]
8	Tauro-alpha-muricholic acid	TaMCA	5.81	514.2	80.0	106.9	123[illegible]
9	Tauro-beta-muricholic acid	TbMCA	5.87	514.2	80.0	106.9	123[illegible]
10	Glycohyocholic acid	GHCA	6.88	464.2	74.0	–	–
11	Trihydroxycholestanoic acid	THCA	6.92	514.2	80.0	106.9	123[illegible]
12	Glycoursodeoxycholic acid	GUDCA	7.10	448.2	74.0	–	–
13	Tauroursodeoxycholic acid	TUDCA	7.11	498.2	80.0	106.9	123[illegible]
14	Glycohyodeoxycholic acid	GHDCA	7.39	448.2	74.0	–	–
15	Taurohyodeoxycholic acid	THDCA	7.40	498.2	80.0	106.9	123[illegible]
16	Perfluoroheptanoic acid	PFHpA	7.53	363.0	169.0	319.0	–
17	7-oxo-deoxycholic acid	7-oxo-DCA	7.61	405.2	123.0	–	–
18	Taurocholic acid	TCA	7.82	514.2	80.0	106.9	123[illegible]
19	Glycocholic acid	GCA	7.84	464.2	74.0	–	–
20	7-oxo-hyocholic acid	7-oxo-HCA	7.88	405.2	375.3	–	–
21	Perfluorohexane sulfonate	PFHxS	7.95	398.9	80.0	98.9	119[illegible]
22	omega/alpha-Muricholic acid	w/a-MCA	7.97	407.2	371.2	–	–
23	beta-Muricholic acid	b-MCA	8.12	407.2	371.2	–	–
24	Perfluorooctanoic acid	PFOA	8.89	413.0	169.0	219.0	369[illegible]
25	Hyocholic acid	HCA	9.11	407.1	389.2	–	–

26	Ursodeoxycholic acid	UDCA	9.25	391.1	391.1	–	–
27	Taurochenodeoxycholic acid	TCDCA	9.26	498.2	80.0	106.9	123[illegible]
28	Glycochenodeoxycholic acid	GCDCA	9.31	448.2	74.0	–	–
29	Taurodeoxycholic acid	TDCA	9.63	498.2	80.0	106.9	123[illegible]
30	Glycodeoxycholic acid	GDCA	9.70	448.2	74.0	–	–
31	Hyodeoxycholic acid	HDCA	9.72	391.1	391.1	–	–
32	Cholic acid	CA	9.74	407.2	343.2	–	–
33	12-oxo-litocholic acid	12-oxo-LCA	9.84	389.1	389.1	–	–
34	Perfluorononanoic acid	PFNA	10.04	463.0	219.0	419.0	–
35	Linear-perfluorooctane sulfonate	L-PFOS	10.27	499.0	80.0	99.0	169[illegible]
36	Taurolitocholic acid	TLCA	10.87	482.2	80.0	106.9	123[illegible]
37	Glycolitocholic acid	GLCA	10.96	432.2	73.9	–	–
38	Chenodeoxycholic acid	CDCA	11.54	391.1	391.1	–	–
39	Deoxycholic acid	DCA	11.78	391.1	391.1	–	–
40	Perfluorodecanoic acid	PFDA	11.04	513.0	219.0	469.0	–
41	Perfluoroundecanoic acid	PFUnDA	11.92	563.0	269.0	519.0	–
42	Perfluorodecane sulfonate	PFDS	12.05	599.0	80.0	98.9	–
43	Perfluorododecanoic acid	PFDoDA	12.70	613.0	169.0	569.0	–
44	Litocholic acid	LCA	13.28	375.1	375.1	–	–
45	Perfluorotridecanoic acid	PFTrDA	13.39	662.9	169.0	619.0	–
Internal standards							
46	[13C4]- Perfluoropentanoic acid	13C4-PFPeA	3.68	266.0	222.0	–	–
47	[13C3]- Perfluoropentanoic acid	13C3-PFBS	4.51	301.9	98.9	–	–
48	[13C2]- Perfluorohexanoic acid	13C2-PFHxA	5.82	315.0	270.0	–	–
49	[D4]- Glycoursodeoxycholic acid	D4-GUDCA	7.09	452.2	74.0	–	–
50	[13C4]- perfluoroheptanoic acid	13C4-PFHpA	7.53	367.0	322.0	–	–
51	[D4]- Taurocholic acid	D4-TCA	7.82	518.2	123.9	–	–
52	[D4]- Glycocholic acid	D4-GCA	7.83	468.2	74.0	–	–
53	[18O3]- perfluorohexane sulfonate	13O3-NaPFHxS	7.95	402.9	102.9	–	–

54	[13C4]- perfluorooctanoic acid	13C4-PFOA	8.89	417.0	372.0	–	–
55	[D4]- Ursodeoxycholic acid	D4-UDCA	9.24	395.1	395.1	–	–
56	[D4]- Glycochenodeoxycholic acid	D4-GCDCA	9.30	452.2	74.0	–	–
57	[D4]- Cholic acid	D4-CA	9.74	411.2	347.2	–	–
58	[13C5]- perfluorononanoic acid	13C5-PFNA	10.04	468.0	423.0	–	–
59	[13C4]- perfluorooctane sulfonate	13C4-NaPFOS	10.27	503.0	99.0	–	–
60	[D4]- Glycolitocholic acid	D4-GLCA	10.96	436.2	73.9	–	–
61	[13C2]- perfluorodecanoic acid	13C2-PFDA	11.04	515.0	470.0	–	–
62	[D4]- Chenodeoxycholic acid	D4-CDCA	11.53	395.1	395.1	–	–
63	[D4]- Deoxycholic acid	D4-DCA	11.78	395.1	395.1	–	–
64	[13C2]- perfluoroundecanoic acid	13C2-PFUnDA	11.92	565.0	520.0	–	–
65	[13C2]- perfluorododecanoic acid	13C2-PFDoDA	12.70	615.0	570.0	–	–
66	[D4]- Litocholic acid	D4-LCA	13.26	379.1	379.1	–	–
Performance standards							
67	[13C5]- perfluoropentanoic acid	13C5-PFPeA	3.68	268.0	223.0	–	–
68	[13C5]- perfluorohexanoic acid	13C5-PFHxA	5.81	318.0	273.0	–	–
69	[13C4]- perfluorohexane sulfonate	13C4-NaPFHxS	7.95	401.9	98.9	–	–
70	[13C8]- perfluorooctanoic acid	13C8-PFOA	8.89	421.0	376.0	–	–
71	[13C9]- perfluorononanoic acid	13C6-PFNA	10.04	472.0	427.0	–	–
72	[13C8]- perfluorooctane sulfonate	13C8-NaPFOS	10.27	507.0	99.0	–	–
73	[13C6]- perfluorodecanoic acid	13C6-PFDA	11.04	519.0	474.0	–	–
74	[13C7]- perfluoroundecanoic acid	13C7-PFUnDA	11.92	570.0	525.0	–	–

Table S3 Recovery mean, recovery range and RSD of the internal standards in the NIST SRM 1957 and QC plasma samples

Internal standards (PFAS)	NIST SRM 1957 (n=4)			QC plasma (n=7)		
	Mean (%)	Recovery range (%)	RSD	Mean (%)	Recovery range (%)	RSD
13C-PFHPeA	119	99.6-120	13.7	111	94.6–127	9.93
13C-PFHxA	111	94.9–116	8.15	106	93.3–122	6.67
13C-PFHpA	118	101–116	6.28	109	81.9–131	13.3
13C-PFHxS	110	89.5–119	11.6	104	92.3–124	7.55
13C-PFOA	110	93.0–115	8.74	106	93.0–121	8.42
13C-PFNA	114	92.4–114	9.28	106	91.5–127	7.97
13C-L-PFOS	116	87.0–116	14.3	107	90.7–128	7.76
13C-PFDA	117	81.5–117	14.7	102	88.8–132	9.53
13C-PFUnDA	119	61.4–117	25.9	90.6	87.2–138	12.1

Table S4 Recovery mean, recovery range and RSD of the bile acids in NIST SRM 1957 and QC plasma samples

Internal standards (BA)	NIST SRM 1957 (n=4)			QC plasma (n=7)		
	Mean (%)	Recovery range (%)	RSD	Mean (%)	Recovery range (%)	RSD
D4-CA	63.9	53.7-83	21%	48.0	37.2-83.1	30%
D4-GCA	52.0	41.4-67.2	21%	55.7	48.8-72.9	13%
D4-GUDCA	70.3	60.2-89.4	19%	70.9	62.2-90.8	13%
D4-GCDCA	48.5	40-61.6	19%	53.4	46.3-77.5	19%
D4-UDCA	100.0	88.5-122.8	16%	75.2	69.8-82.6	6%
D4-CDCA	104.9	87.9-136.1	21%	59.3	50.5-70.5	11%
D4-DCA	81.9	69.2-101.9	17%	52.9	45-65	13%
D4-GLCA	40.2	35.7-48	14%	46.6	39-68	20%
D4-LCA	81.1	67-89.1	12%	74.8	68-90.3	11%

[1]also applied for HCA

[2]also applied for GHCA

Table S5 Linear range, limits of detection, average concentration and RSD of the PFAS and BA in QC plasma samples (n= 10) using 20 µl sample volume

Analyte	r	LLOQ	ULOQ	Average concentration	%RSD
TDHCA	0.9989	0.0025	300	2.07	32.06
TaMCA	0.9982	0.5	300	12.91	6.5
TwMCA	0.9982	1	600		
TbMCA	0.9966	0.25	600	16.76	7.98
GDHCA	0.9991	0.25	600	nd	n/a
THCA	0.9985	0.25	600	25.22	4.52
TUDCA	0.9989	0.25	600	19.74	3.72
7-OXO-DCA	0.999	0.025	600	2.85	43.2
7-OXO-HDCA	0.9986	0.25	600	nd	n/a
aMCA	0.9968	0.5	600	nd	n/a
bMCA	0.9948	1	600	nd	n/a
CA	0.9959	0.25	600	30.62	7.23
CDCA	0.9992	1	600	49.23	5.09
DCA	0.9991	0.025	600	16.55	10.51
DHCA	0.9897	0.5	600	nd	n/a
GCA	0.9992	0.0025	600	317.9	2.49
GCDCA	0.9991	0.0025	600	1004.2	2.02
GDCA	n/a	n/a	n/a		
GHCA	0.9986	0.025	600	34.31	8.45
GHDCA	0.9986	0.0025	600	86.57	4.15
GLCA	0.9998	0.0025	600	3.56	31.3
GUDCA	0.9984	0.0025	600	88.36	3.46
HCA	0.9973	0.25	300	11.31	10.77
HDCA	0.9987	0.5	600	nd	n/a
LCA	0.9989	0.5	300	nd	n/a
TCA	0.9985	1	600	97.97	3.63
TCDCA	0.9997	0.0025	600	380.3	3.55
TDCA	0.9996	0.25	600	12.2	4.96
THDCA	0.9981	0.25	600	14.49	6.8
TLCA	0.993	0.25	600	nd	n/a
UDCA	0.9993	0.25	600	28.52	3.71
wMCA	0.98	10	600	nd	n/a
12-OXO-LCA	0.9982	10	600	nd	n/a
PFBuS	0.9987	0.025	200	nd	n/a
PFDA	0.53	n/a	n/a	n/a	n/a
PFDoDA	0.9995	0.5	200	nd	n/a
PFDS	0.9978	0.5	200	nd	n/a

PFHpA	0.9996	0.025	200	nd	n/a
PFHxA	0.995	0.025	200	nd	n/a
PFHxS	0.9985	0.25	200	nd	n/a
PFNA	0.9993	0.025	200	nd	n/a
PFOA	0.999	0.25	200	n/a*	n/a
PFOS	0.9993	0.025	200	0.53	3.04
PFOSA	0.9997	0.5	200	0.07	7.4
PFPeA	0.9975	0.025	100	nd	n/a
PFTrDA	0.9975	0.5	200	1.57	0.92
PFUnDA	0.9991	10	200	nd	n/a

*High background level in the blanks

Fig. S1 Some BAs such as TCDCA and TDCA as well as TUDCA and THDCA undergoes the same transition (499 > 80 m/z) and also readily to co-elute with L-PFOS. Chromatographic separation and multiple product ions were selected to reduce potential interferences of selected BAs with L-PFOS

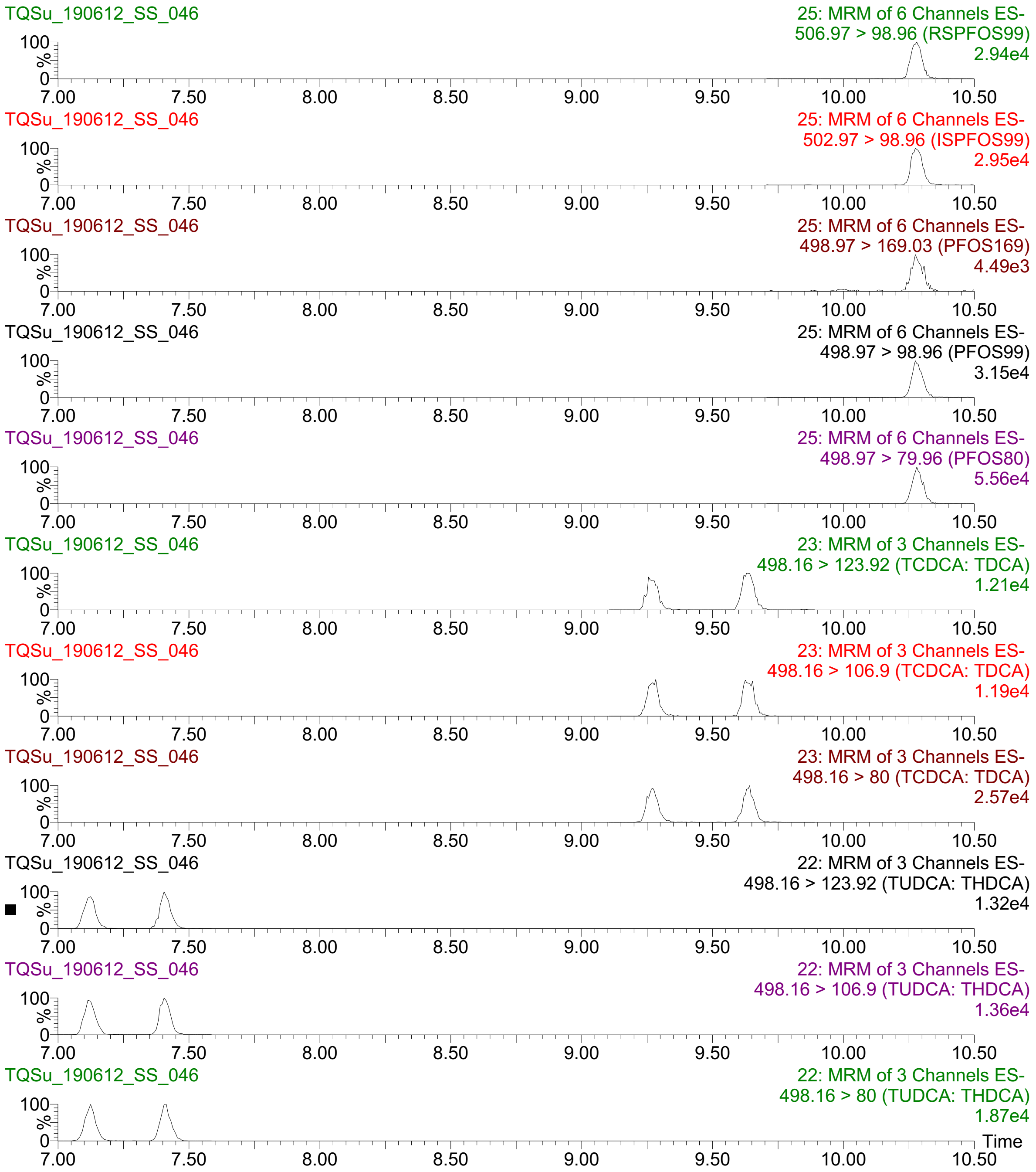

Fig. S2 Matrix suppression for (A) d4-GLCA, (B) d4-GCA and (C) d4-GUDCA, with ISTD added before (upper panels) and after sample clean-up (lower panels) and the deviation between the peak areas

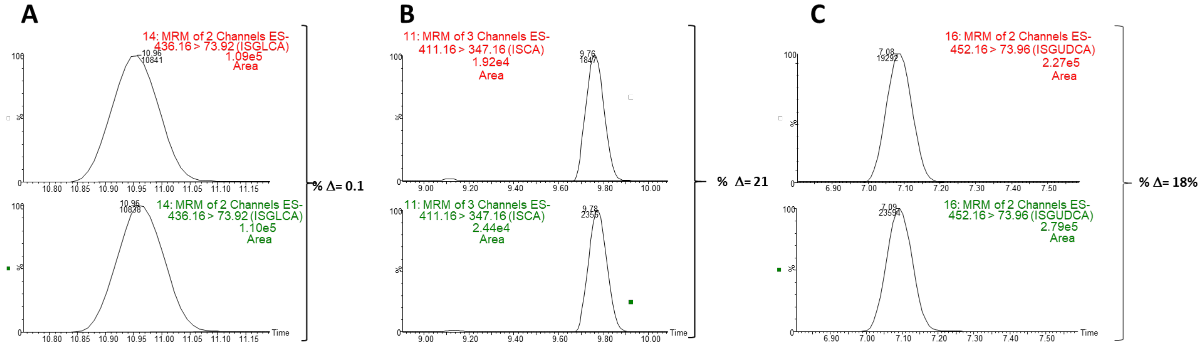

Fig. S3 Boxplots of the measured concentrations of the PFAS and bile acids in the healthy human subjects

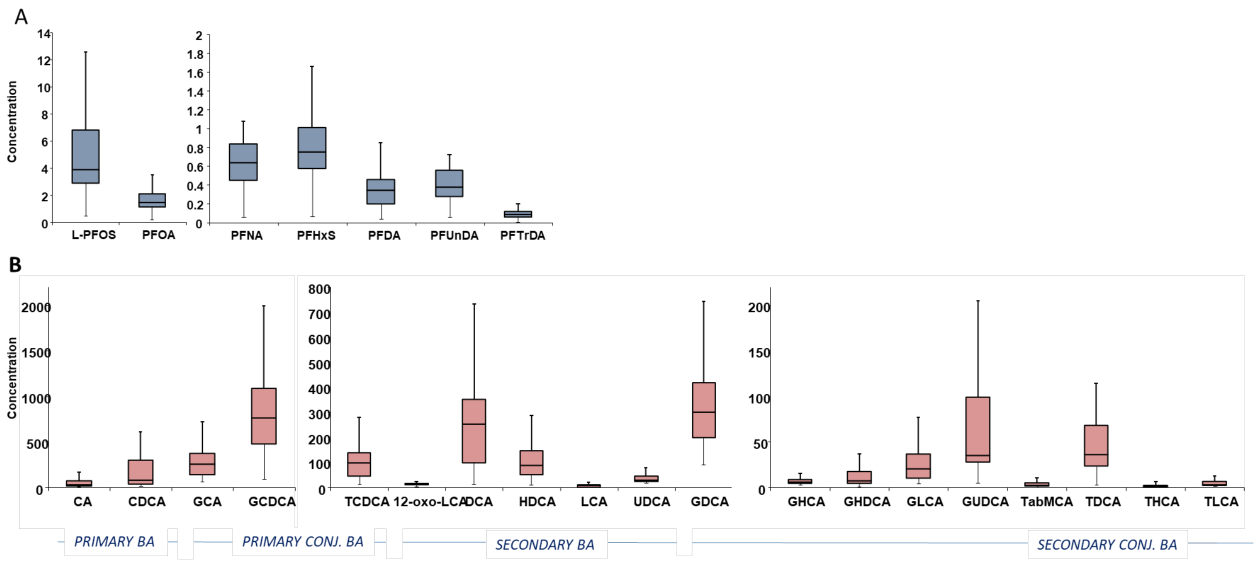

Supplement: Supplementary file 1 — (PDF 419 kb). [file 216_2019_2263_MOESM1_ESM.pdf]
